# Determining cut-off values and predictors for the Snaith–Hamilton Pleasure Scale: comparison between clinical and school settings

**DOI:** 10.1192/bjo.2024.35

**Published:** 2024-05-09

**Authors:** Yen-Chung Ho, Susan Shur-Fen Gau, Ying-Sian Wu, Chun-Hsin Chen, Jiunn-Kae Wang, Hsin-Chien Lee, Kuo-Hsuan Chung, Yi-Hang Chiu, Kah Kheng Goh, Mong-Liang Lu, Yu-Chun Lin, Pi-Chen Chang, Hsiu-Ju Chang

**Affiliations:** School of Nursing, College of Nursing, Taipei Medical University, Taipei, Taiwan; Department of Psychiatry, National Taiwan University Hospital and College of Medicine, Taipei, Taiwan; Graduate Institute of Brain and Mind Sciences, College of Medicine, National Taiwan University, Taipei, Taiwan; and Department of Psychology, National Taipei University, Taipei, Taiwan; School of Nursing, College of Nursing, Taipei Medical University, Taipei, Taiwan; and Department of Nursing, Taipei Veterans General Hospital, Yuli Branches, Hualien, Taiwan; Department of Psychiatry and Psychiatric Research Center, Wan-Fang Hospital, Taipei Medical University, Taipei, Taiwan; and Department of Psychiatry, School of Medicine, College of Medicine, Taipei Medical University, Taipei, Taiwan; Department of Psychiatry, School of Medicine, College of Medicine, Taipei Medical University, Taipei, Taiwan; and Department of Psychiatry, Shuang Ho Hospital, Taipei Medical University, New Taipei, Taiwan; Graduate Institute of Humanities in Medicine, College of Humanities & Social Sciences, Taipei Medical University, Taipei, Taiwan; and Department of Psychiatry & Sleep Center, Taipei Medical University Hospital, Taipei, Taiwan; Department of Psychiatry, School of Medicine, College of Medicine, Taipei Medical University, Taipei, Taiwan; and Department of Psychiatry and Psychiatric Research Center, Taipei Medical University Hospital, Taipei, Taiwan; Department of Psychiatry and Psychiatric Research Center, Wan-Fang Hospital, Taipei Medical University, Taipei, Taiwan; Department of Nursing, College of Nursing, National Yang Ming Chiao Tung University, Taipei City, Taiwan; and Department of Nursing, College of Nursing, Efficient Smart Care Research Center, National Yang Ming Chiao Tung University, Taipei City, Taiwan

**Keywords:** Anhedonia, depression, ROC curve, SHAPS

## Abstract

**Background:**

Few previous studies have established Snaith–Hamilton Pleasure Scale (SHAPS) cut-off values using receiver operating characteristic curve analysis and applied these values to compare predictors of anhedonia between clinical and nonclinical groups.

**Aims:**

To determine the optimal cut-off values for the SHAPS and use them to identify predictors of anhedonia in clinical and nonclinical groups in Taiwan.

**Method:**

This cross-sectional and correlational study used convenience sampling to recruit 160 patients from three hospitals and 412 students from two universities in northern Taiwan. Data analysis included receiver operating characteristic curve, univariate and multivariate analyses.

**Results:**

The optimal SHAPS cut-off values were 29.5 and 23.5 for the clinical and nonclinical groups, respectively. Moreover, two-stage analysis revealed that participants in the clinical group who perceived themselves as nondepressed, and participants in the nonclinical group who did not skip classes and whose fathers exhibited higher levels of care and protection were less likely to attain the cut-off values. Conversely, participants in the nonclinical group who reported lower academic satisfaction and were unwilling to seek help from family or friends were more likely to attain the cut-off values.

**Conclusions:**

Our findings highlight the importance of optimal cut-off values in screening for depression risk within clinical and nonclinical groups. Accordingly, the development of comprehensive, individualised programmes to monitor variation trends in SHAPS scores and relevant predictors of anhedonia across different target populations is crucial.

Depression is a widespread mental disorder affecting 280 million individuals worldwide. It is also a prominent cause of disability and contributes to the global burden of disease.^1^ Experiencing pleasure is essential for an individual's well-being.^2^ However, individuals with mental disorders often have a diminished capacity to experience pleasure.^3^ This inability to experience pleasure is referred to as anhedonia, a term that also describes an individual's lack of response to pleasant stimuli. Anhedonia has profound implications, potentially leading to decreased quality of life.^4,5^ It is among the core symptoms of depression^6^ and is recognised as a transdiagnostic symptom across various mental disorders.^7^ A large population-based study has shown that brain structure is related to phenotypes of and genetic risk factors for anhedonia; however, the study had limitations in terms of access to its target populations.^8^ Therefore, the use of a self-report scale to assess anhedonia is advantageous because it enables researchers to easily reach their target populations. An example of such a scale is the Snaith–Hamilton Pleasure Scale (SHAPS), a 14-item self-report instrument that encompasses four domains of hedonic experience, namely: interest/pastimes, social interaction, sensory experience and food/drink. The scale was developed with consideration of culture, gender and age to minimise potential biases.^9^ The SHAPS has been extensively used for assessing anhedonia in patients with major depressive disorder (MDD) and the general population across different countries and has been demonstrated to exhibit robust psychometric properties.^10^ The scale is also concise and easily comprehensible and has thus been translated into multiple languages, including Spanish, simplified Chinese, Italian and Malay.^11–14^ Despite its widespread application, the SHAPS does not have a standard scoring method. The original scoring method involves the use of a dichotomous scoring system, with 0 indicating agreement and 1 indicating disagreement, and a total score ranging from 0 to 14. However, this scoring system may limit the ability of researchers to distinguish levels of severity for each item, to calculate relevant validity measures and to establish the correlations of SHAPS results with those of other instruments. To address these limitations, researchers have modified the scoring method by adopting a four-point Likert scale with anchors that range from ‘definitely agree’ to ‘definitely disagree,’ yielding a total score ranging from 14 to 56.^9,12,15^ Furthermore, SHAPS reference values have yet to be established because of the lack of a standard scoring method for the scale. In the original version of the SHAPS, individuals are considered to exhibit anhedonia if they disagree with more than two SHAPS items.^9^ A systematic review and meta-analysis of 168 studies reported that the mean reference values for the SHAPS were 20.2 (s.d. = 2.1) and 33.1 (s.d. = 2.7) for nonclinical and clinical groups, respectively.^3^ Nevertheless, the characteristics and severity of anhedonia may vary between clinical and nonclinical groups;^3^ therefore, comparing SHAPS cut-off values between these two groups is imperative. Moreover, studies have shown that anhedonia is a multifaceted symptom that can be influenced by genetic, social and biological factors.^5,16^ Therefore, determining the predictors of anhedonia in both clinical and nonclinical groups is crucial for understanding its underlying mechanism and establishing appropriate intervention and policy measures.

As mentioned above, studies have confirmed a positive correlation between anhedonia and depression^10,12^ and have reported reference values for the SHAPS for both clinical and nonclinical groups.^3^ Nevertheless, few of these studies established SHAPS cut-off values through receiver operating characteristic (ROC) curve analysis or used these values to compare predictors of anhedonia between clinical and nonclinical groups. The Patient Health Questionnaire-9 (PHQ-9) is a reliable, valid, brief and easily administered tool that has been validated for diagnosis of MDD.^17–19^ Accordingly, in the present study, we used the PHQ-9 as a gold standard to determine cut-off values for the SHAPS and then used these values to compare predictors of anhedonia between clinical and nonclinical groups. On the basis of the findings of the aforementioned studies, we hypothesised that a significant correlation would exist between SHAPS and PHQ-9 scores in both clinical and nonclinical groups (hypothesis 1); that SHAPS cut-off values would differ between the clinical and nonclinical groups, with the clinical group having a higher cut-off values (hypothesis 2); and that the prevalence and predictors of high-risk depression, as determined using the SHAPS scores, would differ between the clinical and nonclinical groups categorised according to the optimal cut-off values (hypothesis 3).

## Method

### Research design and study participants

This study employed a cross-sectional and correlational research design. To derive SHAPS cut-off values for use in identifying and comparing predictors of anhedonia between clinical and nonclinical groups, we used convenience sampling to concurrently recruit 160 out-patients with MDD (i.e. the clinical group) from three hospitals and 412 university students (i.e. the nonclinical group) from two universities in northern Taiwan between August 2021 and May 2023. The inclusion criteria for the clinical group were as follows: (a) being aged 18–65 years and an out-patient with MDD; (b) receiving a clinical diagnosis of MDD, confirmed by experienced psychiatrists on the basis of ICD-10 codes (i.e. F32: depressive episode; F33: recurrent depressive disorder; F34: persistent affective disorder; F38: other affective disorder; F39: unspecified affective disorder); (c) being able to communicate in Mandarin Chinese; and (d) being willing to provide informed consent for participation in this study. The inclusion criteria for the nonclinical group were as follows: (a) being a university student; (b) being able to communicate in Mandarin Chinese; and (c) being willing to provide informed consent for participation in this study. Patients with any other psychiatric comorbidity or risk of suicide were excluded from the study. To determine the appropriate sample size for our study, we used G*Power 3.1,^20^ setting the medium effect size to 0.15, significance level to 5% and power to 80%; thus, we derived a total sample size of 118. In addition, we considered the widely accepted recommendation that correlational and comparative research requires a sample size of at least 30 participants in each group.^21^ Thus, our final sample size ensured adequate statistical power for the study.

### Measurements

#### Demographic characteristics

We collected information on the demographic characteristics of the clinical group, including gender, age, height, weight, educational level, geographical area, family status, socioeconomic status, working status, mental-health-related medical status and substance use status. For the nonclinical group, we collected information on demographic characteristics including gender, age, height, weight, geographical area, family status, socioeconomic status, working status, parenting style, available support resources, areas of concern, mental-health-related medical status and substance use status.

#### SHAPS

The SHAPS was applied to assess anhedonia in this study. The SHAPS is a self-report tool comprising 14 items designed to evaluate recent hedonic experiences across four domains: interest/pastimes, social interaction, sensory experience and food/drink.^9^ As mentioned, various language versions of the SHAPS have been developed, including simplified and traditional Chinese versions. The simplified Chinese version of the SHAPS contains items that are rated on a four-point Likert scale with answers ranging from ‘definitely agree’ to ‘definitely disagree’; the total score of this scale ranges from 14 to 56. The test–retest reliability and Cronbach's α value of this scale have been reported to be 0.64 and 0.85, respectively.^12^ Moreover, the traditional Chinese version of the SHAPS is easy to read (*N* = 13, *M* = 8.85) and straightforward to answer (*N* = 13, *M* = 9.23), indicating favourable face validity. The test–retest reliability and Cronbach's α value derived for this scale were 0.87 and 0.91, respectively. Furthermore, the traditional Chinese version of the SHAPS has been reported to demonstrate positive correlations with PHQ-9 (*r* = 0.52, *P* < 0.001) and the Positive and Negative Suicide Ideation – Negative Suicidal Idea (PANSI-NSI) (*r* = 0.28, *P* < 0.01) as well as negative correlations with self-esteem (*r* = −0.51, *P* < 0.001) and the Positive and Negative Suicide Ideation – Positive Idea (PANSI-PI) (*r* = −0.54, *P* < 0.001); therefore, the Chinese version of the SHAPS can be considered to be reliable and valid.^22^

#### PHQ-9

PHQ-9 is a tool designed to detect the presence of depressive symptoms and measure the severity of depression experienced over the preceding 2 weeks. PHQ-9 comprises nine questions, each of which is rated on a four-point scale with end-points ranging from 0 (‘never’) to 3 (‘almost every day’). The total score ranges from 0 to 27, with a score of 5–9 indicating mild depression, 10–14 indicating moderate depression, 15–19 indicating moderately severe depression and ≥20 indicating severe depression.^18,23^ The Chinese version of PHQ-9 has been reported to exhibit good reliability and validity in the Taiwanese population, with an internal consistency of 0.80 for adults and 0.77 for elderly people. In terms of concurrent validity, PHQ-9 exhibited a positive correlation with the Hamilton Rating Scale for Depression (*r* = 0.66, *P* < 0.001) and a negative correlation with the Quality of Life and Satisfaction Survey Volume (*r* = −0.53, *P* < 0.001).^24,25^

### Data collection

As the data collection period overlapped with the COVID-19 pandemic, data from both the clinical and nonclinical groups were collected either online or in person as convenient. For the clinical group, experienced psychiatrists identified potential participants during clinical consultations. Subsequently, research assistants reached out to these potential participants to provide a detailed explanation of the study's purpose and procedures. Those who expressed interest in participating in the study signed informed consent forms and were given the option to complete the research questionnaires either online or in person. For the nonclinical group, prospective students were approached either online or in person during scheduled class times. They were presented with an explanation of the study's purpose and procedures. Those who agreed to participate in the study signed informed consent forms and proceeded to complete the study questionnaires. After completing the questionnaires, all participants received NT$100 vouchers as a token of appreciation for their participation.

### Statistical analysis

Data analysis was conducted using SPSS 24.0. The basic characteristics of the participants are presented as frequencies, percentages, means and s.d. values. Pearson correlation coefficients were derived to evaluate the correlations between the SHAPS and PHQ-9 scores in both the clinical and nonclinical groups. ROC curve analysis was conducted to determine the optimal cut-off values for the area under the ROC curve (AUC) and the sensitivity and specificity of the SHAPS in both the clinical and nonclinical groups. Independent-samples *t*-test, χ^2^-test and multiple logistic regression were used to determine the predictors and determinants of anhedonia on the basis of the SHAPS cut-off values. Furthermore, we conducted a sensitivity analysis to assess the reliability of the ROC curve analysis results in the clinical and nonclinical groups using two approaches. First, the data from both groups were combined and coded as 1 and 0 to replace PHQ-9 as the gold standard for ROC curve analysis. Second, to minimise the potential impact of variations in participant characteristics, SAS 9.4 was used to pair participants in the clinical group with those in the nonclinical group at a 1:2 ratio according to gender, age, height and weight; subsequently, ROC curve analysis was performed, and the results obtained with pairing were compared with those obtained without pairing. Statistical significance was set at 0.05 in this study.

### Ethical considerations

This study protocol was approved by the Joint Institutional Review Board of Taipei Medical University, Taiwan (TMU-JIRB-N202011061) and the Institutional Review Board of National Yang Ming Chiao Tung University, Taiwan (YM110170E). The collected data were pseudonymised to ensure that no personally identifiable information was included. Only the researcher conducting this study had access to these data. Moreover, the data will be securely stored for a minimum of 7 years.

## Results

### Participant characteristics

The nonclinical group comprised 412 students, of whom 67% were women. On the basis of PHQ-9 scores, 174 (42.23%) of the students in the nonclinical group were classified as healthy, 139 (33.74%) were classified as having mild depression, 61 (14.81%) were classified as having moderate depression, 26 (6.31%) were classified as having moderately severe depression and 12 (2.91%) were classified as having severe depression. The mean age, height and weight of students in this group were 21.06 years (s.d. = 1.28; range, 18–25 years), 165.01 cm (s.d. = 7.99; range, 149–186 cm) and 58.52 kg (s.d. = 12.8; range, 36–113 kg), respectively. The clinical group comprised 160 patients, of whom 66% were women. On the basis of PHQ-9 scores, 25 (15.62%) of the patients in this group were classified as healthy, 31 (19.37%) were classified as having mild depression, 32 (20%) were classified as having moderate depression, 33 (20.63%) were classified as having moderately severe depression and 39 (24.38%) were classified as having severe depression. The mean age, height, and weight of patients in the clinical group were 39.49 years (s.d. = 13.91; range, 19–67 years), 163.8 cm (s.d. = 8.48; range, 146–185 cm) and 63.16 kg (s.d. = 15.23; range, 39–123 kg), respectively. We observed significant differences between the two groups in terms of depression levels, age and weight. The baseline characteristics of the participants are presented in Table 1.

**Table 1:** Baseline characteristics of participants in the clinical and nonclinical groups (*N* = 572)

	Clinical group (*N* = 160)		Nonclinical group (*N* = 412)		Test type	*P/*95% CI
	Mean (s.d.)	*N* (%)	Mean (s.d.)	*N* (%)		
Gender					χ^2^	0.921^a^
Male		54 (33.75)		136 (33)		
Female		106 (66.25)		276 (67)		
PHQ-9					χ^2^	<0.001***
Normal		25 (15.62)		174 (42.23)		
Mild		31 (19.37)		139 (33.74)		
Moderate		32 (20)		61 (14.81)		
Moderately severe		33 (20.63)		26 (6.31)		
Severe		39 (24.38)		12 (2.91)		
Age, years	39.49 (13.91)		21.06 (1.28)		*t*	−20.6, −16.25***
Height	163.8 (8.48)		165.01 (7.99)		*t*	−0.28, 2.7
Weight	63.16 (15.23)		58.52 (12.8)		*t*	−7.31, −1.95***

PHQ-9, Patient Health Questionnaire-9.

a. Fisher's exact test.

*** *P* < 0.001.

### Correlations between SHAPS and PHQ-9 scores in clinical and nonclinical groups

Pearson correlation coefficients were computed to assess the linear relationship between SHAPS and PHQ-9 scores in both the clinical and nonclinical groups. The results revealed a positive correlation between the two instruments in both groups (*r* = 0.58, *P* < 0.001 and *r* = 0.41, *P* < 0.001, respectively; Table 2).

**Table 2:** Pearson correlation analysis to assess the linear relationship between SHAPS and PHQ-9 scores in both the clinical and nonclinical groups (*N* = 572)

Scale	Clinical group (*N* = 160)		95% CI	Nonclinical group (*N* = 412)		95% CI
	SHAPS	PHQ-9		SHAPS	PHQ-9	
SHAPS	1	0.58***	0.47, 0.68	1	0.41***	0.32, 0.49
PHQ-9	0.58***	1		0.41***	1	

SHAPS, Snaith–Hamilton Pleasure Scale; PHQ-9, Patient Health Questionnaire-9.

*** *P* < 0.001.

### Comparison of SHAPS cut-off values between clinical and nonclinical groups

**Fig. 1:**
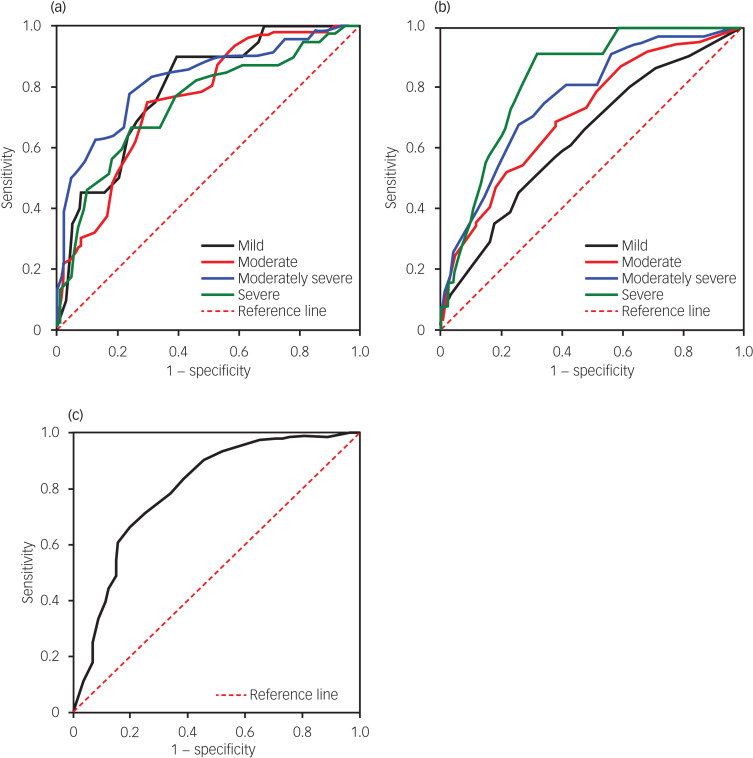
Receiver operating characteristic curves illustrating Snaith–Hamilton Pleasure Scale cut-off values for distinguishing between different severity levels of depression and clinical depression, as derived using Patient Health Questionnaire-9 scores and clinical diagnosis. (a) clinical group, (b) nonclinical group, (c) overall combined group.

We plotted ROC curves to derive SHAPS cut-off values for distinguishing various levels of depression severity, as determined using PHQ-9, in the clinical and nonclinical groups (Fig. 1). The results indicated that in the clinical group, the SHAPS could significantly differentiate between mild, moderate, moderately severe and severe depressive symptoms, with AUCs of 0.79, 0.76, 0.83 and 0.75, respectively (*P* < 0.001). Moreover, the optimal cut-off values corresponding to these levels of depression were determined using the Youden index,^26^ yielding cut-off values of 28.5, 28.5, 29.5 and 32.5 for mild, moderate, moderately severe and severe depressive symptoms, respectively (Table 3). The results also indicated that in the nonclinical group, the SHAPS could significantly distinguish between mild, moderate, moderately severe and severe depressive symptoms, with AUCs of 0.64, 0.72, 0.77 and 0.83, respectively (*P* < 0.001). The Youden index^26^ was again used to determine optimal cut-off values; these were 20.5, 21.28, 24.5 and 23.5 for mild, moderate, moderately severe and severe depressive symptoms, respectively (Table 3). The cut-off value associated with maximum sensitivity (78%) and specificity (76%) for the clinical group was 29.5, whereas that associated with maximum sensitivity (92%) and specificity (68%) for the nonclinical group was 23.5 (Table 3).

**Table 3:** Optimal cut-off values for Snaith–Hamilton Pleasure Scale for the clinical, nonclinical and combined overall groups^a^

	Depressive symptoms														
	Mild				Moderate			Moderately severe					Severe		
Cut-off value	Sensitivity	1 − specificity	Youden index	Cut-off value	Sensitivity	1 − specificity	Youden index	Cut-off value	Sensitivity	1 − specificity	Youden index	Cut-off value	Sensitivity	1 − specificity	Youden index
Clinical group															
13.0000	1.000	1.000	0.000	13.0000	1.000	1.000	0.000	13.0000	1.000	1.000	0.000	13.0000	1.000	1.000	0.000
14.5000	0.971	0.900	0.071	14.5000	0.990	0.911	0.080	14.5000	1.000	0.932	0.068	14.5000	1.000	0.950	0.050
16.0000	0.957	0.750	0.207	16.0000	0.981	0.839	0.141	16.0000	0.986	0.886	0.100	16.0000	0.974	0.917	0.057
17.5000	0.950	0.650	0.300	17.5000	0.981	0.786	0.195	17.5000	0.986	0.852	0.134	17.5000	0.974	0.893	0.082
18.5000	0.929	0.600	0.329	18.5000	0.952	0.768	0.184	18.5000	0.958	0.830	0.129	18.5000	0.949	0.868	0.081
19.5000	0.921	0.550	0.371	19.5000	0.942	0.750	0.192	19.5000	0.958	0.807	0.152	19.5000	0.949	0.851	0.097
21.0000	0.893	0.550	0.343	21.0000	0.923	0.714	0.209	21.0000	0.958	0.761	0.197	21.0000	0.949	0.818	0.131
22.5000	0.886	0.550	0.336	22.5000	0.923	0.696	0.227	22.5000	0.958	0.750	0.208	22.5000	0.949	0.810	0.139
23.5000	0.843	0.550	0.293	23.5000	0.875	0.679	0.196	23.5000	0.917	0.716	0.201	23.5000	0.897	0.777	0.121
24.5000	0.800	0.500	0.300	24.5000	0.837	0.625	0.212	24.5000	0.903	0.648	0.255	24.5000	0.872	0.727	0.145
25.5000	0.764	0.350	0.414	25.5000	0.817	0.518	0.299	25.5000	0.903	0.557	0.346	25.5000	0.872	0.661	0.211
26.5000	0.729	0.300	0.429	26.5000	0.788	0.464	0.324	26.5000	0.889	0.500	0.389	26.5000	0.872	0.612	0.260
27.5000	0.679	0.250	0.429	27.5000	0.750	0.393	0.357	27.5000	0.861	0.432	0.429	27.5000	0.846	0.554	0.292
**28.5000**	**0.607**	**0.100**	**0.507**	**28.5000**	**0.702**	**0.250**	**0.452**	28.5000	0.833	0.307	0.527	28.5000	0.821	0.455	0.366
29.5000	0.536	0.100	0.436	29.5000	0.615	0.232	0.383	**29.5000**	**0.778**	**0.239**	**0.539**	29.5000	0.769	0.388	0.381
30.5000	0.464	0.100	0.364	30.5000	0.529	0.214	0.315	30.5000	0.667	0.216	0.451	30.5000	0.667	0.339	0.328
31.5000	0.429	0.100	0.329	31.5000	0.490	0.196	0.294	31.5000	0.639	0.182	0.457	31.5000	0.667	0.298	0.369
32.5000	0.386	0.100	0.286	32.5000	0.471	0.125	0.346	32.5000	0.625	0.125	0.500	**32.5000**	**0.667**	**0.248**	**0.419**
33.5000	0.336	0.050	0.286	33.5000	0.423	0.071	0.352	33.5000	0.556	0.091	0.465	33.5000	0.590	0.207	0.383
34.5000	0.314	0.000	0.314	34.5000	0.394	0.054	0.341	34.5000	0.528	0.068	0.460	34.5000	0.564	0.182	0.382
35.5000	0.286	0.000	0.286	35.5000	0.365	0.036	0.330	35.5000	0.500	0.045	0.455	35.5000	0.513	0.165	0.348
36.5000	0.214	0.000	0.214	36.5000	0.279	0.018	0.261	36.5000	0.389	0.023	0.366	36.5000	0.462	0.099	0.362
37.5000	0.150	0.000	0.150	37.5000	0.192	0.018	0.174	37.5000	0.264	0.023	0.241	37.5000	0.333	0.066	0.267
38.5000	0.129	0.000	0.129	38.5000	0.163	0.018	0.146	38.5000	0.222	0.023	0.199	38.5000	0.282	0.058	0.224
39.5000	0.093	0.000	0.093	39.5000	0.115	0.018	0.098	39.5000	0.167	0.011	0.155	39.5000	0.179	0.050	0.130
40.5000	0.064	0.000	0.064	40.5000	0.087	0.000	0.087	40.5000	0.125	0.000	0.125	40.5000	0.154	0.025	0.129
42.0000	0.043	0.000	0.043	42.0000	0.058	0.000	0.058	42.0000	0.083	0.000	0.083	42.0000	0.128	0.008	0.120
43.5000	0.036	0.000	0.036	43.5000	0.048	0.000	0.048	43.5000	0.069	0.000	0.069	43.5000	0.103	0.008	0.094
44.5000	0.029	0.000	0.029	44.5000	0.038	0.000	0.038	44.5000	0.056	0.000	0.056	44.5000	0.077	0.008	0.069
46.0000	0.014	0.000	0.014	46.0000	0.019	0.000	0.019	46.0000	0.028	0.000	0.028	46.0000	0.026	0.008	0.017
48.0000	0.007	0.000	0.007	48.0000	0.010	0.000	0.010	48.0000	0.014	0.000	0.014	48.0000	0.026	0.000	0.026
50.0000	0.000	0.000	0.000	50.0000	0.000	0.000	0.000	50.0000	0.000	0.000	0.000	50.0000	0.000	0.000	0.000
Nonclinical group															
13.0000	1.000	1.000	0.000	13.0000	1.000	1.000	0.000	13.0000	1.000	1.000	0.000	13.0000	1.000	1.000	0.000
14.5000	0.912	0.827	0.085	14.5000	0.959	0.860	0.099	14.5000	0.974	0.874	0.099	14.5000	1.000	0.880	0.120
15.5000	0.872	0.712	0.160	15.5000	0.949	0.777	0.172	15.5000	0.974	0.802	0.172	15.5000	1.000	0.813	0.188
16.5000	0.806	0.626	0.180	16.5000	0.929	0.688	0.241	16.5000	0.974	0.722	0.252	16.5000	1.000	0.738	0.263
17.5000	0.725	0.540	0.186	17.5000	0.878	0.596	0.282	17.5000	0.947	0.634	0.314	17.5000	1.000	0.653	0.348
18.3485	0.667	0.475	0.192	18.3485	0.816	0.535	0.281	18.3485	0.921	0.570	0.352	18.3485	1.000	0.590	0.410
18.8485	0.663	0.475	0.188	18.8485	0.816	0.532	0.284	18.8485	0.921	0.567	0.354	18.8485	1.000	0.588	0.413
19.5000	0.612	0.424	0.187	19.5000	0.745	0.487	0.258	19.5000	0.816	0.521	0.294	19.5000	0.917	0.538	0.379
**20.5000**	**0.575**	**0.374**	**0.201**	20.5000	0.724	0.439	0.285	20.5000	0.816	0.476	0.340	20.5000	0.917	0.495	0.422
21.2788	0.524	0.324	0.200	**21.2788**	**0.694**	**0.382**	**0.312**	21.2788	0.816	0.420	0.396	21.2788	0.917	0.443	0.474
21.7788	0.520	0.324	0.196	21.7788	0.684	0.382	0.302	21.7788	0.816	0.417	0.399	21.7788	0.917	0.440	0.477
22.5000	0.458	0.259	0.199	22.5000	0.612	0.322	0.291	22.5000	0.763	0.353	0.410	22.5000	0.917	0.375	0.542
23.5000	0.396	0.230	0.165	23.5000	0.551	0.274	0.277	23.5000	0.711	0.302	0.408	**23.5000**	**0.917**	**0.323**	**0.594**
24.5000	0.355	0.180	0.175	24.5000	0.531	0.223	0.308	**24.5000**	**0.684**	**0.257**	**0.428**	24.5000	0.833	0.280	0.553
25.5000	0.300	0.165	0.135	25.5000	0.480	0.185	0.295	25.5000	0.605	0.219	0.386	25.5000	0.750	0.240	0.510
26.5000	0.267	0.144	0.124	26.5000	0.418	0.166	0.253	26.5000	0.553	0.193	0.360	26.5000	0.667	0.213	0.454
27.0173	0.212	0.101	0.112	27.0173	0.357	0.118	0.239	27.0173	0.447	0.147	0.300	27.0173	0.583	0.163	0.421
27.5173	0.209	0.101	0.108	27.5173	0.347	0.118	0.229	27.5173	0.447	0.144	0.303	27.5173	0.583	0.160	0.423
28.5000	0.125	0.036	0.089	28.5000	0.245	0.048	0.197	28.5000	0.316	0.072	0.244	28.5000	0.333	0.088	0.246
29.2329	0.092	0.014	0.077	29.2329	0.184	0.029	0.155	29.2329	0.263	0.045	0.218	29.2329	0.250	0.060	0.190
29.7329	0.088	0.014	0.074	29.7329	0.184	0.025	0.158	29.7329	0.263	0.043	0.220	29.7329	0.250	0.058	0.193
30.5000	0.062	0.007	0.055	30.5000	0.133	0.016	0.117	30.5000	0.184	0.029	0.155	30.5000	0.167	0.040	0.127
31.5000	0.048	0.007	0.040	31.5000	0.092	0.016	0.076	31.5000	0.158	0.021	0.137	31.5000	0.167	0.030	0.137
32.5000	0.033	0.007	0.026	32.5000	0.061	0.013	0.048	32.5000	0.132	0.013	0.118	32.5000	0.083	0.023	0.061
33.5000	0.026	0.007	0.018	33.5000	0.051	0.010	0.041	33.5000	0.105	0.011	0.095	33.5000	0.083	0.018	0.066
34.5000	0.022	0.007	0.015	34.5000	0.041	0.010	0.031	34.5000	0.079	0.011	0.068	34.5000	0.083	0.015	0.068
35.5000	0.018	0.000	0.018	35.5000	0.041	0.003	0.038	35.5000	0.079	0.005	0.074	35.5000	0.083	0.010	0.073
37.5000	0.015	0.000	0.015	37.5000	0.041	0.000	0.041	37.5000	0.079	0.003	0.076	37.5000	0.083	0.007	0.076
39.5000	0.011	0.000	0.011	39.5000	0.031	0.000	0.031	39.5000	0.053	0.003	0.050	39.5000	0.083	0.005	0.078
40.5000	0.007	0.000	0.007	40.5000	0.020	0.000	0.020	40.5000	0.053	0.000	0.053	40.5000	0.083	0.002	0.081
46.0000	0.004	0.000	0.004	46.0000	0.010	0.000	0.010	46.0000	0.026	0.000	0.026	46.0000	0.083	0.000	0.083
52.0000	0.000	0.000	0.000	52.0000	0.000	0.000	0.000	52.0000	0.000	0.000	0.000	52.0000	0.000	0.000	0.000
	Clinical depression														
Cut-off value	Sensitivity	1 − specificity	Youden index												
Overall combined group															
13.0000	1.000	1.000	0.000												
14.5000	0.963	0.883	0.079												
15.5000	0.931	0.818	0.113												
16.5000	0.931	0.745	0.186												
17.5000	0.913	0.663	0.250												
18.3485	0.888	0.602	0.286												
18.8485	0.888	0.600	0.288												
19.5000	0.875	0.549	0.326												
20.5000	0.850	0.507	0.343												
21.2788	0.850	0.456	0.394												
21.7788	0.850	0.454	0.396												
22.5000	0.844	0.391	0.453												
**23.5000**	**0.806**	**0.340**	**0.466**												
**24.5000**	**0.763**	**0.296**	**0.466**												
25.5000	0.713	0.255	0.458												
26.5000	0.675	0.226	0.449												
27.0173	0.625	0.175	0.450												
27.5173	0.625	0.172	0.453												
28.5000	0.544	0.095	0.449												
29.2329	0.481	0.066	0.416												
29.7329	0.481	0.063	0.418												
30.5000	0.419	0.044	0.375												
31.5000	0.388	0.034	0.354												
32.5000	0.350	0.024	0.326												
33.5000	0.300	0.019	0.281												
34.5000	0.275	0.017	0.258												
35.5000	0.250	0.012	0.238												
36.5000	0.188	0.010	0.178												
37.5000	0.131	0.010	0.122												
38.5000	0.113	0.010	0.103												
39.5000	0.081	0.007	0.074												
40.5000	0.056	0.005	0.051												
42.0000	0.038	0.002	0.035												
43.5000	0.031	0.002	0.029												
44.5000	0.025	0.002	0.023												
46.0000	0.013	0.002	0.010												
48.0000	0.006	0.002	0.004												
50.0000	0.000	0.002	−0.002												
52.0000	0.000	0.000	0.000												

a. Optimal cut-off values based on the Youden index are presented in bold.

### Sensitivity analysis

First, all participants in the clinical group received their diagnoses from experienced psychiatrists through structured clinical interviews, a widely accepted gold standard for psychiatric diagnoses.^27^ Accordingly, we conducted a sensitivity analysis to ascertain the reliability of our ROC curve analysis. For this analysis, we combined data from both the clinical and nonclinical groups and assigned them binary codes of 1 and 0 as substitutes for PHQ-9, which is the gold standard. Subsequently, we generated ROC curves to establish SHAPS cut-off values to effectively distinguish depression, as diagnosed clinically, in the overall combined group (Fig. 1). In the combined group, SHAPS demonstrated significant discriminatory ability for clinical depression, with a corresponding AUC of 0.8 (*P* < 0.001). Moreover, the optimal cut-off values for clinical depression were determined using the Youden index.^26^ The optimal cut-off values for clinical depression were identified as 23.5 with a sensitivity of 81% and specificity of 66%, and 24.5 with a sensitivity of 76% and specificity of 70%, in the overall combined group (Table 3). These results were consistent with the optimal cut-off values determined for the nonclinical group.

Second, we paired the participants in the clinical group with those in the nonclinical group at a 1:2 ratio and then conducted ROC curve analysis. The characteristics of the participants in the clinical and nonclinical groups did not differ significantly when the participants were paired according to gender (*P* = 1), age (*P* = 0.3), height (*P* = 0.61) or and weight (*P* = 0.13). Moreover, the ROC curve analysis results obtained for these paired groups were consistent with those obtained for the original groups.

### Prevalence and predictors of high-risk depression based on optimal SHAPS cut-off values in both clinical and nonclinical groups

We used the optimal SHAPS cut-off values to estimate the prevalence of high-risk depression in the two groups. The estimated prevalence of high-risk depression was 48.1% in the clinical group and 34% in the nonclinical group. Furthermore, we showed by chi-squared test that high-risk depression was significantly correlated with self-awareness of depression *(P* = 0.002) and self-harm history *(P* = 0.03) in the clinical group. An independent-samples *t*-test also indicated that high-risk depression was significantly correlated with lower academic performance *(P* < 0.001) and academic satisfaction *(P* < 0.001) scores in the nonclinical group. In addition, chi-squared test results demonstrated that in the nonclinical group, high-risk depression was significantly correlated with skipping classes *(P* < 0.001); father's parenting attitude (*P* = 0.01); mother's parenting attitude (*P* = 0.02); help-seeking behaviours, including seeking help from relatives (*P* < 0.001) and friends (*P* = 0.006); receiving psychological counselling (*P* = 0.012); and seeing a psychiatrist (*P* = 0.002; Table 4).

**Table 4:** Predictors of high-risk depression based on optimal cut-off values for Snaith–Hamilton Pleasure Scale

	Clinical group					
	Low-risk group		High-risk group		Test type	*P*
	Mean (s.d.)	*N* (%)	Mean (s.d.)	*N* (%)		
Self-awareness of depression					χ^2^	0.002
Yes		59 (71.1)		59 (76.6)		
No		14 (16.9)		1 (1.3)		
Not sure		10 (12.0)		17 (22.1)		
Self-harm history					χ^2^	0.03
Yes		25 (30.0)		36 (46.8)		
No		58 (70.0)		41 (53.2)		

	Nonclinical group					
	Low-risk group		High-risk group		Test type	*P*
	Mean (s.d.)	*N* (%)	Mean (s.d.)	*N* (%)		
Academic performance	2.65 (0.74)	272 (66.0)	2.98 (0.79)	140 (34.0)	*t*	<0.001
Academic satisfaction	2.63 (0.79)	272 (66.0)	2.97 (0.77)	140 (34.0)	*t*	<0.001
Skipping classes					χ^2^	<0.001
>75%		110 (40.0)		39 (27.9)		
50–74%		117 (43.0)		56 (40.0)		
25–49%		37 (14.0)		32 (22.9)		
<25%		7 (2.6)		7 (5.0)		
Never		1 (0.4)		6 (4.2)		
Parenting attitude						
Father's parenting attitude					χ^2^	0.01
High care, high protection		111 (41.2)		43 (31.6)		
High care, low protection		58 (21.6)		22 (16.2)		
Low care, high protection		69 (25.7)		38 (27.9)		
Low care, low protection		31 (11.5)		33 (24.3)		
Mother's parenting attitude					χ^2^	0.02
High care, high protection		169 (62.1)		76 (55.1)		
High care, low protection		66 (24.3)		25 (18.1)		
Low care, low protection		24 (8.8)		22 (15.9)		
Low care, low protection		13 (4.8)		15 (10.9)		
Help-seeking-behaviours					χ^2^	<0.001
Seeking help from relatives						
Yes		130 (47.8)		37 (26.4)		
No		142 (52.2)		103 (73.6)		
Seeking help from friends					χ^2^	0.006
Yes		206 (75.7)		88 (62.9)		
No		66 (24.3)		52 (37.1)		
Psychological counselling					χ^2^	0.012
Yes		60 (22.1)		47 (33.6)		
No		212 (77.9)		93 (66.4)		
Seeing a psychiatrist					χ^2^	0.002
Yes		17 (6.3)		22 (15.7)		
No		253 (93.7)		118 (84.3)		

We finally conducted multiple logistic regression analyses, including factors such as self-awareness of depression and self-harm history for the clinical group and factors such as academic performance, academic satisfaction, skipping classes, parenting attitude, help-seeking behaviours, receiving psychological counselling and seeing a psychiatrist for the nonclinical group. These factors were included because they differed significantly between participants at high and low risk of depression in both groups (Table 4).

In the clinical group, compared with the participants with a low risk of depression, those who believed that they were not depressed (odds ratio = 0.08; 95% CI 0.01–0.64) had a lower probability of having a high risk of depression. In the nonclinical group, compared with participants with a low risk of depression, those who had lower academic satisfaction (odds ratio = 1.55; 95% CI 1.09–2.21) and were not willing to seek help from relatives (odds ratio = 1.80; 95% CI 1.09–2.96) or friends (odds ratio = 1.79; 95% CI 1.09–2.96) had a higher probability of having a higher risk of depression. Moreover, participants whose fathers’ parenting attitudes were characterised by greater care and protection (odds ratio = 0.4; 95% CI 0.18–0.89) and those who were not skipping classes (odds ratio = 0.48; 95% CI 0.25–0.9) had a lower probability of having a high risk of depression (Table 5).

**Table 5:** Determinants of high-risk depression based on the optimal cut-off values for the Snaith–Hamilton Pleasure Scale

	Clinical group		
		High-risk depression group	
	*B*	Odds ratio	(95% CI)
Self-awareness of depression			
Not sure	0.629	1.877	0.781–4.511
No	−2.524	0.080*	0.010–0.635
Yes	Ref.		
Self-harm history			
No	−0.674	0.510	0.259–1.004
Yes	Ref.		

	Nonclinical group		
		High-risk depression group	
	*B*	Odds ratio	(95% CI)
School related factors			
Academic satisfaction	0.438	1.550*	1.085-2.213
Academic performance	0.112	1.119	0.770–1.627
Skipping classes			
Never	−0.742	0.476*	0.252–0.899
>25%	Ref.		
Help-seeking behaviours			
Seeking help from relatives			
No	0.585	1.795*	1.089–2.957
Yes	Ref.		
Seeking help from friends			
No	0.583	1.791*	1.085–2.957
Yes	Ref.		
Psychological counselling			
No	−0.231	0.793	0.448–1.405
Yes	Ref.		
Seeing a psychiatrist			
No	−0.613	0.542	0.230–1.277
Yes	Ref.		
Parenting attitude			
Father's parenting attitude			
High care, high protection	−0.913	0.401*	0.182–0.886
High care, low protection	−0.774	0.461	0.200–1.061
Low care, high protection	−0.766	0.465	0.212–1.018
Low care, low protection	Ref.		
Mother's parenting attitude			
High care, high protection	0.071	1.074	0.368–3.132
High care, low protection	−0.380	0.684	0.227–2.062
Low care, high protection	0.257	1.292	0.397–4.203
Low care, low protection	Ref.		

Ref., reference group (low-risk depression group).

* *P* < 0.05.

## Discussion

To the best of our knowledge, this is the first study to establish SHAPS cut-off values for both clinical and nonclinical populations using ROC curve analysis. We calculated Pearson correlation coefficients to assess the correlation between SHAPS and PHQ-9 scores and used ROC curve analysis to determine optimal SHAPS cut-off values for both clinical and nonclinical groups. Our findings demonstrate a significant correlation between SHAPS and PHQ-9 scores. Moreover, the optimal SHAPS cut-off values derived in this study could effectively distinguish between individuals at risk of depression in both the clinical and nonclinical groups.

We observed a significant correlation between SHAPS and PHQ-9 scores in both the clinical and nonclinical groups; thus, hypothesis 1 is supported. This finding is also consistent with those of previous studies.^10,12^ The correlation coefficient for the association between SHAPS and PHQ-9 scores in the clinical group was higher (moderate positive) than that in the nonclinical group (moderate positive). Furthermore, we noted that the optimal SHAPS cut-off values differed between the clinical and nonclinical groups, with the clinical group having higher cut-off values; thus, hypothesis 2 is supported. This finding is consistent with those of previous research.^3^ The differences in cut-off values may be attributed to variations in the severity of anhedonia and baseline depression levels between the clinical and nonclinical groups. Studies have demonstrated that anhedonia is more prevalent in patients with MDD (up to 70%)^28^ than in the general population (approximately 20%).^29^ PHQ-9 is widely used to evaluate the severity of depression, whereas the SHAPS is used to assess anhedonia, a key symptom of depression.^12,24,25^ Therefore, clinical populations may have higher SHAPS scores and a stronger correlation with anhedonia when compared with nonclinical populations; this is consistent with our findings (Table 1) and those of a previous study.^10^

The optimal SHAPS cut-off values derived in this study could help to determine the prevalence and predictors of high-risk depression in clinical and nonclinical groups; thus, hypothesis 3 is supported by our results. The optimal SHAPS cut-off was 23.5 in the nonclinical group; on the basis of this cut-off value, we determined that the prevalence of high-risk depression was 34% in this group. This is consistent with the findings of the Global Point Prevalence Survey for elevated depressive symptoms and with the results of a population-based cohort study.^30,31^ Accordingly, the optimal SHAPS cut-off (23.5) derived for the nonclinical group has the potential to distinguish high-risk individuals from a target population. Furthermore, the optimal SHAPS cut-off was 29.5 in the clinical group; on the basis of this cut-off value, we determined that the prevalence of high-risk depression was 48.1% in this group. This finding is consistent with those of previous studies, which have demonstrated that nearly 50% of patients with MDD did not experience major improvements after routine treatment, and that the recurrence rate of MDD after the first episode was approximately 50%.^32,33^ Hence, the optimal SHAPS cut-off (29.5) derived in this study for the clinical group could serve as an early indicator of poor treatment response or recurrence risk in individuals with MDD. In addition, our logistic regression results show that participants who perceived that they were not depressed exhibited more favourable outcomes compared with those who perceived that they were depressed. This finding raises questions about the role of patient belief and self-awareness of depression. Therefore, we subsequently analysed the correlation between self-awareness of depression and emotional conditions on the basis of the optimal SHAPS cut-off values derived for the two groups. The results revealed that 70% of participants who had scores that exceeded the optimal cut-off values believed that they were depressed, whereas 93% of participants who did not meet the criteria believed that they were not depressed. The significant correlation between self-awareness of depression and emotional condition suggests that patient insight plays a crucial part in the patient's recognition and acknowledgement of their emotional state. Therefore, we confirmed that a substantial proportion of the participants with depression had a reasonable awareness of their condition. This finding aligns with those of previous research, which has reported that participants with MDD exhibited better insight into their conditions, and that 36.8% of the participants had impaired insight into their conditions.^34,35^ The results thus indicate that the optimal cut-off value established for the clinical group could be a valuable tool to monitor the risk of recurrence in patients with poor insight into their depression. Furthermore, our logistic regression model identified risk factors for depression in the nonclinical group, including lower academic satisfaction and poor help-seeking behaviours. These findings are consistent with those of previous research, which demonstrated a significant correlation between lower academic satisfaction and depression (β = −0.26, *P* < 0.001);^36^ they are also consistent with the results of a large-scale national survey that revealed a significant correlation between decreased help-seeking behaviours and depressive symptoms.^37^ Moreover, the model showed protective factors against depression in the nonclinical group, including father's parenting attitude and good attendance at school. These results are also consistent with those of previous studies, which have emphasised the importance of fathers’ involvement and support, as well as regular school attendance, in reducing depressive symptoms.^38,39^ Our findings underscore the significance of the father's parenting attitude in mitigating depression in the nonclinical group. The absence of significance regarding the mother's parenting attitude in the final model may be attributed to cultural factors prevalent in Asian parenting. Research has demonstrated that the authoritarian parenting style, characterised by coldness, lack of support and stringent control, is associated with lower resilience and a higher risk of depression.^40^ Furthermore, traditional Chinese fathers typically wield more power and authority over their children than mothers.^41^ Consequently, the observed importance of high-care and high-protection parenting attitudes of fathers in this study suggests that such attitudes, in contrast to the authoritarian style, may act as a significant protective factor against depression in nonclinical populations.

Our findings reveal a significant correlation between SHAPS and PHQ-9 scores. In addition, we derived optimal SHAPS cut-off values for the clinical and nonclinical groups and then used these values to identify risk factors for high-risk depression in both groups. Notably, these cut-off values and risk factors differed between the clinical and nonclinical groups; therefore, developing strategies and interventions tailored to either group could help to enhance the effectiveness of primary and secondary preventive measures. For example, routine health education programmes could be implemented across diverse populations to help educate them on how to use the SHAPS for self-reflection and to document emotional changes, which could ultimately facilitate the implementation of primary preventive measures. Moreover, the study highlights the potential of SHAPS and its optimal cut-off values as early indicators of depression risk, especially in clinical settings. Identifying individuals at high risk of recurrence or poor treatment response could enable the provision of timely interventions and support. Intervention programmes should also consider both group-level patterns (optimal SHAPS cut-off values) and individual-level changes in SHAPS scores to maximise their effectiveness. Furthermore, the risk factors identified in this study could serve as event markers for emotional support and crisis intervention. Monitoring and intervention programmes should be tailored to an individual's characteristics. For example, individuals at high risk of recurrence may benefit from more intensive support and relapse-prevention strategies, whereas those in nonclinical settings may require educational interventions.

This study had several strengths. First, we concurrently recruited participants from two schools and three hospitals across different districts in Taiwan to explore SHAPS cut-offs and the correlations between SHAPS and PHQ-9 scores in both clinical and nonclinical groups, which increased the external validity of our findings. Second, the inclusion of data from both clinical and nonclinical groups allowed us to conduct clinical discrimination, derive optimal SHAPS cut-off values and use these values to identify predictors of anhedonia. Third, in contrast to most studies in the literature, which have focused on developing and validating SHAPS items without establishing or comparing SHAPS cut-off values within clinical and nonclinical groups, the present study employed PHQ-9 as a gold standard to determine the optimal SHAPS cut-off values and subsequently identify relevant risk factors within the clinical and nonclinical groups. In addition, we employed clinical and nonclinical categories (coded as 1 and 0) as the gold standard instead of PHQ-9 to validate the feasibility of the derived cut-off values. The results not only confirmed the feasibility of the derived cut-off values but also demonstrated the validity of PHQ-9 as a gold standard.

This study also had some limitations that should be acknowledged. First, we adopted a cross-sectional research design. Therefore, we could not fully establish causal relationships between risk factors and SHAPS scores. Second, although we conducted multiple sensitivity analyses to validate our results, the effects of participant characteristics must be considered. Accordingly, our findings should be interpreted with caution, and longitudinal studies with multicentre sampling should be conducted in the future. Finally, the SHAPS is not a diagnostic instrument but a screening tool for identifying individuals at high risk of depression or recurrence. Therefore, individuals whose SHAPS scores reach an established cut-off value should undergo further assessment and evaluation and should receive early interventions, if necessary.

This study confirms that there is a significant correlation between SHAPS and PHQ-9 scores, highlighting the utility of the SHAPS as a tool for assessing anhedonia in individuals. In addition, the optimal SHAPS cut-off values and the predictors of anhedonia determined using these values differed between the clinical and nonclinical groups. These findings demonstrate that such optimal cut-off values and predictors can be useful in the screening of individuals at high risk of depression or recurrence. Our findings study also emphasise the need for comprehensive, individualised programmes using smart devices to screen for anhedonia. These programmes could use SHAPS scores and relevant predictors to identify at-risk individuals and provide early interventions. Furthermore, future research could benefit from longitudinal follow-up studies that monitor SHAPS scores and related predictors over time. This approach would enable a deeper understanding of the dynamic nature of anhedonia and provide opportunities for timely interventions.

## Data Availability

The data that support the findings of this study are available from the corresponding author, H.-J.C., upon reasonable request.
